# The impact of chronic pain on adolescents and their families: A qualitative investigation of parental perspectives

**DOI:** 10.1080/24740527.2025.2562440

**Published:** 2025-12-08

**Authors:** Lindsay Sullivan, Ellie Ferguson, Harrison Vriese, Kathleen Lemanek, Lindsey Vater, Sharon Wrona, Lauren Renner, Megan Armstrong, Hannah Williams, Henry Xiang

**Affiliations:** a Division of Health Sciences, School of Health and Rehabilitation Sciences, College of Medicine, The Ohio State University, Columbus, Ohio, USA; b Department of Pediatrics, The Ohio State University College of Medicine, Columbus, Ohio, USA; c Department of Pediatric Psychology & Neuropsychology, Nationwide Children’s Hospital, Columbus, Ohio, USA; d Comprehensive Pain & Palliative Care Services, Nationwide Children’s Hospital, Columbus, Ohio, USA; e Center for Injury Research and Policy, The Abigail Wexner Research Institute at Nationwide Children’s Hospital, Columbus, Ohio, USA; f Center for Pediatric Trauma Research, The Abigail Wexner Research Institute at Nationwide Children’s Hospital, Columbus, Ohio, USA

**Keywords:** Chronic pain, adolescents, parents, qualitative, experiences

## Abstract

**Background:**

Understanding the experiences of parents of adolescents with chronic pain is crucial in creating a better experience for all involved throughout the adolescent’s chronic pain journey. However, limited qualitative research has explored the experiences of parents of adolescents with chronic pain.

**Aims:**

This qualitative study explored the lived experiences of parents of adolescents with chronic pain, with a focus on the impact of chronic pain on their child and family life. Methods: We conducted 12 semi-structured interviews with parents of adolescents with chronic pain receiving care through a pain management program. Data were transcribed and analyzed using inductive thematic analysis.

**Results:**

Seven key themes were generated and divided into two groups: (1) adolescent and (2) family (including both parents and siblings). Groups were determined based on whether the theme referred to the effect of chronic pain on the adolescent or the caregiver or family. The adolescent group included four themes: (1) physical, (2) psychological, (3) social interaction, and (4) school functioning. The family group included three themes: (1) disruption to daily life, (2) emotional, and (3) relationship dynamics.

**Conclusions:**

This study provides a deeper understanding of the negative effect chronic pain can have on adolescents and family life. Our findings call for interventions to mitigate the physical, psychological, and social impact of chronic pain on adolescents. Family level interventions are also needed to support families of adolescents with chronic pain. More research is needed to explore adolescents’ own views of their experiences with chronic pain.

## RÉSUMÉ

Il est essentiel d’approfondir la compréhension des expériences vécues par les parents d’adolescents atteints de douleur chronique, afin d’améliorer l’expérience de l’ensemble des personnes impliquées tout au long du parcours lié à; la douleur chronique. À ce jour, toutefois, très peu d’études qualitatives se sont penchées sur cette perspective parentale. Objectifs : Cette étude qualitative a exploré les expériences vécues par les parents d’adolescents atteints de douleur chronique, en portant une attention particulière à; l’impact de cette affection sur la vie de leur enfant et sur leur vie familiale.

**Méthodes**: Nous avons mené 12 entretiens semi-structurés auprès de parents d’adolescents atteints de douleur chronique suivis dans un programme spécialisé de prise en charge de la douleur. Les données ont été transcrites et analysées à; l’aide d’une analyse thématique inductive.

**Résultats**: Sept thèmes clés ont été dégagés et regroupés en deux grandes catégories : (1) l’adolescent et (2) la famille (incluant les parents et les frères et sœurs). Cette classification reposait sur la portée du thème, selon qu’il faisait référence à; l’effet de la douleur chronique sur l’adolescent ou sur le ou les proches aidants et la famille. Le groupe « adolescent » comprenait quatre thèmes : (1) physique, (2) psychologique, (3) interactions sociales et (4) fonctionnement scolaire. Le groupe « famille » comprenait trois thèmes : (1) perturbations de la vie quotidienne, (2) émotionnel et (3) dynamique relationnelle.

**Conclusions**: Cette étude contribue à; une meilleure compréhension des effets de la douleur chronique sur les adolescents et la vie familiale. Les résultats soulignent la nécessité de mettre en place des interventions visant à; atténuer l’impact physique, psychologique et social de cette affection chez les adolescents. Des interventions ciblant la sphère familiale sont également nécessaires afin de soutenir les familles d’adolescents atteints de douleurs chroniques. Enfin, des recherches supplémentaires sont nécessaires pour explorer le point de vue des adolescents eux-mêmes sur leur expérience de la douleur chronique.

## Introduction

Chronic pain—defined as pain persisting for longer than 3 months—is a complex, debilitating condition that can significantly interfere with daily life and affect an individual’s quality of life and well-being.^1,^^2^ It is estimated that about one in five children or adolescents (age ≤ 19 years) experience some form of chronic pain.^3^ The prevalence of chronic pain in children and adolescents varies by demographic characteristics, with girls and those in their early teen years significantly more likely to report chronic pain than their counterparts.^3^ Although chronic pain can be caused by an injury or ongoing or progressive disease, in many cases, the source of pain is nonspecific or unexplained.^4,5^ The most common types of chronic pain experienced by children or adolescents are headaches (up to 83%), abdominal pain (up to 53%), and musculoskeletal pain (up to 36%).^3,5^ Children or adolescents can experience a spectrum of pain acuity, with the intensity, duration, and impact of chronic pain varying from child/adolescent to child/adolescent.^6,7^ Some children or adolescents with chronic pain experience mild discomfort, whereas others experience debilitating pain that significantly affects their daily lives.^6,7^ Early and proactive management of chronic pain in children and adolescents is essential in reducing pain intensity and frequency and improving quality of life, productivity, and one’s ability to perform activities of daily living.^8^

The current standard for chronic pain management in children or adolescents is a multidisciplinary approach to address the physical, social, and psychological aspects of pain.^9,10^ This multidisciplinary approach often includes medical management, physical or occupational therapy to improve functioning, and psychological interventions to enhance coping skills and mental health and well-being.^11^ Despite strong evidence supporting the importance of multidisciplinary approaches to chronic pain management in children or adolescents,^12^ many families encounter significant challenges when these treatment plans are introduced. One major barrier is logistical access to specialized pain management programs. Specialized pain management programs are generally located in urban areas, making access difficult for families who reside in rural or socially disadvantaged areas.^13^ Further, specialized pain management programs operate during standard business hours, which can make participation difficult due to conflicting patient’s school or caregiver’s work schedules.^14–16^ Another major barrier to accessing consistent and comprehensive care is the high cost of multidisciplinary pain treatments, especially for families with limited insurance coverage or high-premium insurance plans.^13^ Families of children with chronic pain often face out-of-pocket expenses in providing health care for their child, reduced work participation, and substantial time demands managing their child’s health care needs.^17,18^ These barriers to accessing high-quality pain care, such as limited insurance plans, are region specific. These logistical and financial barriers may exacerbate disparities in chronic pain care.^19^

Chronic pain can significantly disrupt adolescents’ daily life by limiting their physical functioning, including their ability to perform activities of daily living and participate in sports or recreational activities.^20^ It can also negatively affect their mental and social well-being,^21–23^ contributing to reduced engagement in social activities and subsequent social isolation and withdrawal.^24^ This stress and isolation can contribute to the development or exacerbation of mental health problems such as symptoms of anxiety or depression.^25^ Experiencing chronic pain can also lead to missed school days and diminished academic and cognitive performance.^26–28^

Chronic pain not only affects the individual with pain; it also impacts family life.^29–31^ Parenting a child or an adolescent with chronic pain can significantly affect parents’ emotional well-being, contributing to elevated stress levels and depressive symptoms, parental guilt, feelings of helplessness or frustration, and burnout or exhaustion.^31–33^ Chronic pain can also significantly interfere with the family’s social activities due to necessary adjustments to their daily routines, schedules, and interactions.^34,35^ Additionally, parents of children with chronic pain may neglect their own needs and self-care, with some parents losing their sense of identity outside of their role as a caregiver.^17,36^ Chronic pain often imposes a significant financial burden on parents and caregivers, often requiring time off from work or even job loss due to frequent medical appointments or missed school days.^17^

Although chronic pain could have a significant impact on patients and families, very few qualitative studies exploring the impact of chronic pain on family life are published.^31,37^ One qualitative study investigated the impact of caring for a child with chronic pain and found that it impacts various aspects of life, including work, finances, social life, partner relationships, and psychological and emotional functioning.^31^ Another qualitative study explored fathers’ experiences of parenting children with chronic pain, with findings suggesting that fathers often adopt several unique roles to minimize the impact of adolescent chronic pain on their child, self, and family.^37^ However, to the best of our knowledge, little attention has been focused on parents’ perceptions of their child’s experiences with chronic pain or the impact of chronic pain on the wider family network.

Recent qualitative work has taken a closer look at what parents experience while managing a child’s chronic pain and has uncovered a set of recurring themes. Across many pain diagnoses, caregivers describe a cycle of intense feelings, including anxiety, fear, shock, and helplessness, that often arises during the search for a diagnosis and returns whenever symptoms increase, as reported in studies of juvenile idiopathic arthritis where families felt caught off guard by the illness.^38,39^ The ripple effects on everyday life are also clear: employment, finances, household routines, partner relationships, and sibling attention often suffer, and parents speak of feeling powerless while trying to keep multiple roles balanced, observations that appear in work on neuropathic pain and functional abdominal pain.^40–42^ Many families recount persistent obstacles within the health system, noting disappointment with approaches that seem counterintuitive, limited guidance from professionals, and a longing for clinicians to acknowledge their child’s distress.^43–45^ Even so, several studies point to pockets of resilience: tighter family bonds, personal growth, and the benefits of peer communities or therapeutic programs such as acceptance and commitment therapy that give parents and children a common vocabulary for coping.^46–48^ At the same time, caregivers draw attention to their own depleted well-being and shifting parental identities and argue that only truly holistic, family-centered care can soften these burdens.^31,35,49–52^

Though previous qualitative studies have provided valuable insights into the experiences of parents of adolescents with chronic pain, much of this literature is now dated and may not reflect the current landscape of pediatric and adolescent pain care. Over the past 2 decades, our understanding of adolescent chronic pain has deepened, and its management has evolved, marked by the broader adoption of multidisciplinary programs and the systematic integration of psychological and social support services.^10–12^ The health care context has also shifted, with policy changes, new access pathways (including telehealth), and the growing operationalization of the biopsychosocial model of pain.^53–55^ At the same time, societal change and technological advances have altered adolescents’ lives and family dynamics. For instance, the digital revolution has redefined adolescents’ social environments and self-concepts, creating novel challenges for parents in supporting their child’s holistic development. Contemporary qualitative research is therefore needed to capture the present-day experiences and perspectives of parents of adolescents with chronic pain. This study addresses this need by providing an updated and comprehensive account of the impact of chronic pain on adolescents and their families, grounded in today’s models of pediatric and adolescent pain care and health care context. Our findings will provide critical evidence for improving the provision of patient-centered care for adolescents with chronic pain and therefore help optimize physical and psychosocial health treatment outcomes.

## Methods

### Study design and procedure

The current study is part of a larger qualitative study exploring the lived experiences of being a parent of an adolescent with chronic pain and recommendations for innovative at-home interventions to help adolescents effectively manage their chronic pain.^56^ We used a qualitative descriptive design to gain a better understanding of the impact of chronic pain on adolescents with chronic pain and their families. Qualitative descriptive studies aim to provide a detailed and comprehensive description of participants’ lived experiences or perspectives.^57–59^ This approach, which is grounded in naturalistic inquiry, is particularly well suited for research questions that focus on enhancing our understanding of phenomena without imposing a theoretical or interpretive framework.^57–59^ Qualitative description has been increasingly applied in pain research, especially when the goal is to inform clinical practice.^60–64^

### Sampling and recruitment of participants

We employed purposive sampling to recruit participants who could provide rich and relevant insights aligned with the study’s aims. Parents were eligible for the study if they were English-speaking, and they had a child aged 10 to 17 years old who was receiving comprehensive care for chronic pain (defined as pain for a period of at least three months) at the time of study enrollment. Recruitment of participants took place through the Comprehensive Pain Services Department at a large Midwest children’s hospital, after ethical approval was obtained. A range of chronic pain acuity and functional impairments are represented as participants were specifically recruited through either a bi-weekly outpatient program or a 3-week intensive outpatient program.

We applied Malterud’s concept of information power to guide decisions about sample size and composition.^65^ According to this framework, the adequacy of a qualitative sample is determined by the richness and relevance of the data in relation to the study’s aim as opposed to the number of participants alone.^65^ Given the clarity and focus of our research question, the targeted nature of our sample, the depth of the data collected during interviews, and our analytic strategy, we determined that our sample size was sufficient to yield meaningful insights.

### Procedure

Eligible parents were introduced to the study by a member of their child’s health care team during a program visit. Those who were interested in participating in the study were given an information sheet containing important details about the study aims, methods, and time commitment. The information sheet included a QR code that directed interested individuals to REDCap where they could review the consent form, provide informed consent, and complete the demographics survey. As part of the demographics survey, participants were asked to provide their e-mail address, which was used by the research team to follow up and schedule telephone interviews. Only those who voluntarily completed the consent process and provided their contact information were included in the study. Parents were also recruited in waiting areas during a follow-up visit. Parents recruited in person participated in the interview in a private room, after completing the online consent form and demographics survey on a research computer. We interviewed one parent per family, except for one case where both parents wanted to participate; the family designated which parent would participate. We conducted interviews between March and November 2024. Seven interviews were conducted via telephone, and five interviews were conducted in person in a private room at the pain management clinic. Interviews were audio-recorded and lasted between 13 and 65 min (mean = 35 min 15s). Interviews conducted in person tended to be longer and more in-depth. All interviews were transcribed verbatim and deidentified for analysis. The institutional review board (IRB) at The Ohio State University reviewed this study and determined it exempt with limited IRB review (2023E1333).

### Materials

A semistructured interview guide was developed by the research team based on a review of existing literature and consultation with pain researchers and clinicians. The interview guide included a series of open-ended questions and probe questions, allowing the interviewer to follow the parents’ narratives. Therefore, though some questions were asked of all parents, other questions evolved out of the conversation between the interviewer and participant and focused on understanding each participant’s unique experiences. The interview guide explored four broad areas: (1) history of chronic pain (e.g., “Tell me a little bit about when your child first started having chronic pain,” (2) experiences with chronic pain (e.g., “Tell me a little bit about your child’s experiences since they first started having chronic pain,” (3) management of chronic pain (e.g., “How is your child’s chronic pain treated?”), and (4) virtual reality technologies for chronic pain management (e.g., “If you were to design a virtual reality program for adolescents with chronic pain, what would it look like?”). For the purposes of this study, only the qualitative information on experiences with chronic pain are presented. Other data obtained from this study were published elsewhere.^56^ Demographic data about the participating parent and their child with chronic pain were collected prior to the interview through a brief online survey.

### Reflexivity and positionality

In qualitative research it is important to acknowledge that the research teams’ backgrounds, experiences, identities, and perspectives influence interactions with study participants and the interpretation of data.^66^ The current study was conducted by a diverse team of researchers and clinicians, including nurse practitioners, psychologists, and academic scholars with backgrounds in child and adolescent health and qualitative methodologies. Our varied professional experiences enriched the research process by bringing multiple perspectives to the design, data collection, and interpretation phases of this study. We acknowledge that our clinical and academic roles may have influenced how we engaged with participants and interpreted the data. We therefore took deliberate steps to center participants’ voices in our findings. To mitigate potential bias and ensure the integrity of our analysis, we engaged in ongoing reflexive practices throughout the research process, including team discussions and critical reflection on our assumptions and positionalities. Our use of qualitative descriptive methodology supported our commitment to presenting participants’ experiences in a way that is grounded in their own voices and minimally shaped by theoretical frameworks.

### Data analysis

Data were analyzed thematically using an inductive approach.^67^ Inductive approaches for qualitative data analysis provide a convenient and efficient way of analyzing data to gain a holistic understanding of what was said and to ensure that all important aspects of the data are captured.^68,69^ We analyzed the data in three steps: (1) immersion in the topic, (2) identification of possible themes, and (3) reviewing of themes. First, two coders (L.S. and E.F.) read each transcript to familiarize themselves with the data and note their initial impressions. Second, the coders reread the transcripts and independently annotated them line by line, generating initial codes relevant to the research question and broadly related to parents’ perceptions of the impact of chronic pain on their child and family life. The coders discussed the generated codes and developed a codebook with themes and definitions so that coding was consistent across interviews and coders. All interviews were then coded by L.S. and E.F. using the codebook as a guide. The coders met regularly to test the completeness of themes that had been generated and discuss discrepancies. Discrepancies were resolved through discussion or input from another member of our research team (H.V.). This process continued until no new themes were generated to ensure a comprehensive and shared understanding of the experiences shared by parents. Data saturation was achieved after 12 interviews, aligning with prior qualitative studies highlighting that saturation typically occurs within 10 to 12 interviews.^70,71^ To ensure that findings were grounded in evidence, quotations were recorded for each theme. Additionally, we used a code–recode strategy,^72^ peer debriefing,^72,73^ reflexive journaling,^73^ and purposeful sampling to establish the credibility and trustworthiness of our data.

The findings reported here answer the following key research questions: (1) How do parents perceive the impact of chronic pain on their child? and (2) How do parents of adolescents with chronic pain describe its impact on family life? We organized the themes under the broader categories of “adolescent” and “family” to enhance clarity and coherence in presenting our findings. This structure was informed by the interview guide, which included two distinct questions including one focused on the experiences of their child with chronic pain and one on their personal experiences along with the broader family’s experiences. These thematic groupings also emerged inductively from the data, reflecting how participants described their experiences in relation to their child and other family members. This organizational approach aligns with qualitative descriptive methodology, which supports grouping themes to reflect meaningful contextual dimensions and improve accessibility of findings.^74^

## Results

We interviewed 13 parents of adolescents aged 10 to 17 years old (mean = 15.0, SD = 2.49) with chronic pain. Most parents were mothers (*n* = 10, 76.9%) of daughters (*n* = 9, 75.0%) with chronic pain. The mean duration of the adolescent’s chronic pain was 2.87 years (SD = 2.22). Pain complaints differed across adolescents, with most adolescents experiencing multiple pain complaints (*n* = 10, 83.3%) such as headaches including migraine (*n* = 8, 66.7%) and/or joint pain (*n* = 6, 50.0%). Demographic characteristics of participating parents and their children with chronic pain are shown in Table 1.

**Table 1.** Demographic characteristics of participating parents and their child with chronic pain.

**Parent**	
Sex	
Female	76.9 (10)
Male	23.1 (3)
Age, mean (SD), years	44.93 (5.85)
**Child**	
Sex	
Female	75.0 (9)
Male	25.0 (3)
Age, mean (SD), years	15.47 (2.42)
Duration of chronic pain, mean (SD), years	2.87 (2.22)
Type of chronic pain (select all that apply)	
Abdominal	30.8 (4)
Arm	16.7 (2)
Back	41.7 (5)
Body	33.3 (4)
Chest	25.0 (3)
Headaches, including migraine	66.7 (8)
Joint	50.0 (6)
Leg	41.7 (5)
Neck	25.0 (3)
Pelvic	25.0 (3)

The analysis of the interview data provided important insights into the significant impact of chronic pain on adolescents and their families. Seven key themes were generated and organized under the broader categories of “adolescent” or “family,” depending on whether they described the impact of chronic pain on the adolescent or their family. The first group encompassed the influence of chronic pain on the adolescent with chronic pain and included four themes: (1) physical, (2) psychological, (3) social interaction, and (4) school functioning. The second group, family, encompassed the influence of chronic pain on the family, including both parents and siblings, and included three themes: (1) disruption to daily life, (2) emotional, and (3) relationship dynamics. These themes are described in detail in the following sections (Table 2).

**Table 2.** Parents’ perspectives regarding the impact of chronic pain on their child and family life.

Subtheme	Quotations
Impact of chronic pain on children	
Psychological	“He’s very an anxious person now. Seems like it’s gotten worse. And he’s very angry when he gets to where he gets hurting. So, I think that that’s like overhyped everything. And, uh, yeah, I think that’s made him a different person.” (Parent 7, female)
	“When they start out young, they don’t realize that they’re different. … Get older. She’s starting to realize, like, hey, nobody else is feeling the same pain I am. ‘Why am I feeling this?’ Yeah, the anger sets in. … The, you know, ‘Why me? Why is this me? Why can’t they just fix it as in their surgery? Isn’t there a magic pill? Why do I gotta take all these pills?’” (Parent 11, female)
Physical	“Um, with her pain I would have to say. Affected her social. Life for great. Her mental health. Her physical health. Oh my God, it’s back to the whole world. Her whole life is gone. … That’s hard for a teenager.” (Parent 5, female)
	“It was headaches and stomach pains a lot. … The headaches were getting worse and there, like, really wasn’t an explanation. It was just trying to throw medicine at it.” (Parent 9, female)
Social	“And just crying because you just wanted to go to school. She just wanted to be around her friends, or she’s missing out on things because she doesn’t feel good.” (Parent 9, female)
	“She wouldn’t do anything with friends because she just felt like she couldn’t keep up. She didn’t feel good. She didn’t want to. … All of last spring, she really didn’t do anything well, like go to the—you know, they’d go out after—after concert or after event. They go out for, you know, Frosty’s at Wendy’s and she wouldn’t go.” (Parent 10, female)
School	“He’s missed school, he’s laid in pain for hours at home. … When he first started it three years ago, he was missing weeks at a time. Lately it’s just a day here and there. He unfortunately just deals with it.” (Parent 4, female)
	“She missed, like, months at a time. … I had to get a lawyer involved. … They were really nasty to her … we’re still in a court battle because of their nastiness.” (Parent 5, female)
Impact of chronic pain on family	
Emotional	“Yeah, I think it’s impacted me more like, I guess, internally, like the feeling of helplessness and wanting to fix it has been probably the biggest obstacle or difficulty for me.” (Parent 1, female)
	“I’ve felt pretty helpless, and it didn’t feel very fair—sorry—that my kid is hurting so bad that she can’t, you know, be a proper kid.” (Parent 2, female)
Financial	“It’s time-consuming and obviously costly. … So, I think that’s why his pediatrician referred him to the pain clinic to see if maybe they could try some new things and help get this under control.” (Parent 4, female)
	“Our insurance didn’t cover acupuncture. … I’ve been calling some local places … no one wants to work on a minor.” (Parent 6, female)
Relationship dynamics	“Well, if we didn’t know at first what this was like, we just thought, like, there’s this confusion within the household that, like, well, this is not my child. Like, what’s going on here?” (Parent 8, male)
	“I think it’s significantly impacted her relationship with her brother because she just became really intolerant of him. … And they used to be really close.” (Parent 10, female)
Disruption to daily life	“Even going for walks and stuff like that … she couldn’t walk for any length of time without being like, “Oh, my goodness, I hurt so bad.” (Parent 2, female)
	“We spent like nine days at [the hospital], and I also have little kids, so it was a lot. … We had to switch to online school just because she had a lot of trouble finishing a school day.” (Parent 3, female)

### Impact of chronic pain on adolescents

#### Psychological

Many parents spoke of the negative impact of chronic pain on their child’s psychological health and well-being due to the intense physical, emotional, and social challenges it presents. Parents highlighted how their child’s chronic pain contributed to symptoms of anxiety or depression, low quality of life, or negative self-esteem, which generally impacted various aspects of their child’s life.

There was a lot of depression that we were dealing with, ‘Is this going to be the rest of my life? Am I going to just hurt for the rest of my life? (Parent 2, female)

Parents described how their child’s chronic pain provoked a range of emotions, including anger, frustration, sadness, loss of control, loneliness, or isolation, with one parent noting that *“it’s an emotional roller coaster”*(Parent 5, female). Parents discussed how their child’s inability to engage in physical or social activities due to their pain or fatigue contributed to and often exacerbated this range of emotions. Parents also described how difficult it was for their children to accept that they, unlike their peers, were experiencing persistent pain that inhibited their physical and social functioning. A number of parents acknowledged this sentiment by sharing that their child would frequently question, “Why me?” This may reflect an emotional struggle with the unfairness and unpredictability of their pain and a search for meaning and control in their situation. Supporting children through these thoughts is essential for promoting resilience and psychological well-being. Moreover, a few parents talked about the dramatic changes they witnessed in their child after they started experiencing chronic pain, highlighting how their child became a different person after they started experiencing pain. It is essential to address the psychological health and well-being of children with chronic pain because it can improve coping, resilience, and long-term outcomes. A parent recalled the changes she witnessed in her child:

You see this switch in her with the pain and line the light is dimmed (Parent 9, female).

#### Physical

Several parents described how their child’s persistent pain limited their physical functioning, preventing their child from engaging in physical and recreational activities that they once enjoyed. Parents described their child’s symptoms as barriers to engagement in physical and recreational activities, either because they prevented participation in such activities or because the child feared exacerbating their symptoms. Addressing these barriers is essential because physical activity may promote symptom management, resilience, and long-term health and well-being in children and adolescents.

Even going for walks and stuff like that … she couldn’t walk for any length of time without being like, “Oh, my goodness, I hurt so bad.” (Parent 2, female)

Other parents shared how their child’s symptoms, such as headaches and stomach pain, were difficult to manage and worsened over time. These symptoms can disrupt daily functioning, limit school participation, and strain social relationships, which may lead to increased social withdrawal, mental health challenges, and developmental delays.

Her primary and really only symptom was a headache that never goes away and basically does not respond to any treatment that we have tried. … She has not had a headache-free hour or day since over two years ago. (Parent 1, female)

A few parents highlighted how limited physical functioning negatively influenced their child’s mental health and well-being, increasing levels of stress, anxiety, and depression, as well as lowering self-esteem. Several parents noted that there are often no visible, external signs to indicate that their child is physically suffering from chronic pain, making it difficult for others to understand what their child is going through.

#### Social interaction

It was evident that chronic pain can have a profound negative impact on adolescent’s social functioning and relationships, with several parents indicating that the greatest toll impacted by chronic pain is on peer and familiar relationships.

So, social life is what it affects the most. Yeah, because in high school, if you’re not there and people in your friends faces every day, especially in a big school, yeah, they forget you. (Parent 11, female)

Many parents highlighted how their child’s persistent pain led to social withdrawal, isolation, or feeling like they “can’t keep up” with their peers, which, in turn, exacerbated symptoms of anxiety, depression, or low self-worth. Several parents discussed how the invisibility of chronic pain often contributes to a lack of understanding among others, including their peers and teachers, leading to frustration and a sense of invalidation. To protect themselves from feeling different or judged, these children may begin to avoid social situations, which in turn can disrupt emotional development, weaken peer relationships, and increase the risk of mental health problems such as anxiety or depression.

I think to some degree it has impacted her relationships because … it’s very much an invisible thing for her, and I think it’s hard from that perspective of not really like getting any … there’s not really an understanding. (Parent 1, female)

Another parent highlighted how chronic pain significantly impacted their child’s relationship with their sibling, causing a rift in a once strong relationship. More specifically, this parent highlighted how the increased focus on the child with chronic pain and restrictions on family activities caused resentment and the child without chronic pain feeling overlooked or neglected. They also noted that the child was no longer able to engage in many of the activities that they used to enjoy doing with their siblings, resulting in a loss of shared interests.

#### School functioning

Parents described the substantial influence of chronic pain on their child’s school functioning, including their academics and quality of school life. Due to chronic pain, many children frequently missed school, with several parents highlighting that their child “was missing weeks at a time” (Parent 4, female) or in some cases “missed, like, months at a time” (Parent 5, female). Extended absences can significantly disrupt a child’s development by limiting opportunities for academic learning, social interaction, and emotional growth. A few parents emphasized the emotional toll of missing school on their child.

The past school year has been the worst. I don’t even know if she actually ever went to school for a full week. … And just crying because she just wanted to go to school. (Parent 9, female)

Other parents described how their child’s chronic pain sometimes impacted their ability to complete their homework, which resulted in them falling behind in class and negatively influenced their academic performance. The pressure to catch up academically may also be overwhelming and anxiety-provoking for children with chronic pain. Several parents highlighted a lack of support from school officials, including teachers being unwilling or hesitant to implement academic accommodations or support strategies, leading to frustration, barriers in accessing education, and in some cases legal battles. One parent shared how their child had to switch to online school due to academic and school-related problems stemming from their child’s chronic pain, emphasizing how this in turn negatively impacted their child’s social relationships by limiting opportunities for daily, in-person interaction. This lack of peer connection may make it difficult for children with chronic pain to maintain friendships or develop new ones, hindering their social development and well-being. It may also make reintegrating back into school more stressful and difficult to navigate.

So, we had to switch to online school just because she had a lot of trouble finishing a school day. And then it affects her social life, like, she’s 16 but she doesn’t do a lot of stuff with friends and stuff like that. (Parent 3, female)

Another parent discussed how their child had difficulties physically getting around school during the day due to their pain and symptoms. This was largely due to the school being a multistory building and the adolescent having to travel long distances to get from one class to another. Others shared that attending school exacerbated their child’s pain symptoms. Although attending school offers a sense of normalcy for children with chronic pain, it appears that the demands of school life may intensify physical pain and emotional distress in some children and adolescents.

### Impact of child chronic pain on the family

#### Disruption to daily life

All parents reported that their everyday lives were disrupted by their child’s chronic pain. Some parents expressed how they had to take family medical leave or quit their jobs to take care of their children. Parents expressed how taking leave from work or quitting their job to take care of their child caused significant financial stress for their family, adding another layer of emotional distress for parents who already feel overwhelmed about their child’s chronic pain.

I actually ended up taking medical leave for all of last spring to take care of her and him [talking about another child who was sick]. (Parent 10, female)

I had to quit my job. … And I don’t regret it. Yeah, that’s just how it was. (Parent 11, female)

A few parents discussed how although intensive pain management programs can positively impact quality of life and return to daily activities, they can also contribute to increased stress and anxiety in families, especially those with other younger children, by requiring frequent trips to the outpatient program or hospital. The structured pain program that many of the children of participating parents completed was 5 days a week, 8 h a day, over a 3-week period. Many of the families noted that they had to travel long distances for their child to attend the outpatient program, with some families traveling from other states, due to the lack of high-quality pain care near their homes. The need to travel long distances for care required some families to source temporary housing close to the outpatient care setting. Some parents also highlighted financial hardship due to the costs associated with pain management programs and services and with some treatments (e.g., acupuncture, massage) not being covered by insurance companies. These out-of-pocket expenses may be especially burdensome for families who must travel long distances for specialized care, leading to missed work, reduced income, and being away from other children or family members.

It affects your finances very much. … I had to quit my job. … It’s just financially a lot coming to the hospital, … eating out, parking, you know. (Parent 11, female)

#### Emotional

Parents highlighted the emotional impact of their child’s chronic pain on them and other members of their family. Many parents described feelings of helplessness, frustration, sadness, and guilt. Parents expressed feeling powerless due to their inability to alleviate their child’s physical pain and psychological distress despite their strong desire to protect and comfort their child. The uncertainty of whether, and when, their child would get better exacerbated feelings of helplessness, frustration, and sadness. Some parents also expressed guilt and self-blame for not identifying the source of their child’s pain earlier, delayed treatment, or being unable to prevent their child’s pain. These feelings of guilt and self-blame negatively impacted the emotional well-being of parents.

It’s almost been devastating … the mental toll it has taken on my husband and I, watching her go through this. … It just messes with your head so bad. … You’re just helpless watching your kids suffer. (Parent 9, female)

Another parent shared:

I mean, it’s heartbreaking because I just—I want to fix him, and I don’t know how. We just go day by day. … It’s hard as the mother to know your child is in pain and you can’t fix it. (Parent 4, female)

For several parents, one of the hardest aspects of their child’s experience with chronic pain was seeing their once-healthy child unable to participate in activities that they once enjoyed or that other adolescents can experience without pain, leading to feelings of frustration or sadness. These parents appeared to be grieving the loss of a typical childhood for their sons/daughters, because chronic pain often leads to prolonged school absences and limited opportunities for peer interaction and carefree exploration. These parents expressed how their child’s chronic pain not only was causing emotional distress and social isolation but was robbing their children of the freedom to simply experience childhood and adolescence.

#### Relationship dynamics

Many parents highlighted how their child’s chronic pain influenced the quality of their interpersonal relationships. A few parents described how their relationship with their child was deeply affected by the onset of their child’s chronic pain. These parents discussed how the psychological challenges their child faced, such as or depression symptoms, social withdrawal, or isolation, led to behavioral changes including emotional reactivity, which strained the parent–child bond. These parents emphasized the importance of parental education to better one’s understanding of how to communicate and relate to their child with chronic pain and to help them effectively navigate the many challenges experienced by their children. For example, parents discussed how they were instructed not to ask their child about their chronic pain on a regular basis, which they found counterintuitive. Parents also discussed how their child’s chronic pain affected their relationship with their spouse or partner, often resulting in an emotional and psychological burden and contributing to poor mental health and strained relationships. This was largely due to the stressful circumstances generated by the child’s illness and the need to change or modify their family roles to cope with the emotional and practice demands of caring for a child with chronic pain. Further, it was common for the primary caregiver of the child with chronic pain to report investing most of their physical and emotional energy into their child’s chronic pain care, reducing time and emotional availability for their partner and contributing to communication breakdowns and conflicts. One parent acknowledged this sentiment by saying:

My husband, you know, … he just goes in the other room because he can’t hear it. … He doesn’t like to deal with it. So, he just goes in the other room and lets me deal with it, which is a tough burden to carry. (Parent 7, female)

## Discussion

Our findings showed that chronic pain often has a profound negative impact on the lives of adolescents and their families. Parents discussed the substantial impact of chronic pain on their son’s or daughter’s physical, psychological, social, and school functioning. They highlighted how the invisibility of chronic pain makes it challenging for others to understand what their child is going through, contributing to social withdrawal and symptoms of anxiety and depression. Parents also emphasized the profound emotional, mental, and financial burden of chronic pain on the family. These findings have important implications for adolescent chronic pain care, underscoring the need for comprehensive strategies and resources to help families navigate the physical, psychological, and social aspects of chronic pain and to enhance the overall experiences of these adolescents and their families.

Findings from this study support previous quantitative work that indicates that chronic pain affects various aspects of child and family life.^35^ Similar to previous research,^75,76^ our results indicate that chronic pain significantly affects physical, psychological, social, and school functioning in adolescents. Many parents discussed how their child’s chronic pain hindered their participation in physical and social activities, including their ability to maintain or pursue hobbies and meet friends because of their pain. Parents described how limitations in functioning and activities of daily living often contributed to worse quality of life, social withdrawal and isolation, and elevated symptoms of anxiety and depression in their children. Past research shows that physical activity and exercise may help reduce pain severity and improve the physical functioning of adolescents with a wide variety of chronic pain conditions, such as chronic neck pain,^77^ headache,^78–80^ and fibromyalgia.^81,82^ Parents in our study also discussed how their child’s chronic pain resulted in a significant amount of missed school days. These findings are in line with prior studies on chronic pain in adolescents.^24,83^ Taken together, our findings emphasize the importance of pain management plans that are individualized and address the wide-ranging impact of chronic pain on adolescents by focusing on pain control, function, and social and emotional well-being.

In our study, parents emphasized that the invisibility of chronic pain often leads to misunderstandings with friends, teachers, and even doctors, because some individuals do not recognize the everyday challenges their child experiences due to their chronic pain. This misunderstanding can result in emotional isolation, strained personal relationships, social withdrawal, and poor mental health and well-being in adolescents with chronic pain. It can even prevent adolescents with chronic pain from getting appropriate academic accommodations, which may negatively impact school functioning and school self-concept. Studies show that various situational factors, such as the presence of documented medical evidence, direct communication from the medical team, and parental attitudes, influence teachers’ responses to students with pain.^84,85^ Assessing how schools respond to pain problems in their students is essential in ensuring implementation of accommodations and supporting students’ mental health and emotional well-being. Prior studies also show the influence of peer responses to adolescents’ pain symptoms, with high-quality peer relationships contributing to adaptive responses to chronic pain.^86,87^ It is unclear to what extent pediatric chronic pain programs provide a medical explanation of an adolescent’s chronic pain to teachers and peers that affect how pain is perceived and managed in the school setting by fostering empathy, understanding, and support.

The findings from this study suggest that chronic pain significantly affects family life. Parents described how their child’s chronic pain contributed to disruptions in daily life, elevated emotional distress, and weakened interpersonal relationships. Some parents discussed how they had to quit their job to help care for their child with chronic pain, which led to financial hardship. Many parents emphasized the emotional distress they experienced because of their inability to help their child. Specifically, they felt ineffective at helping with the physical, emotional, and behavioral changes they observed in their child, as well as changes to their personality, self-worth, and self-efficacy. These findings are in line with published studies^31,37^ and may be shaped by cultural and historical understandings of childhood and adolescence. Ngo et al.^31^ found wide-reaching impacts of chronic pain on family life due to a variety of factors, including the constant nature of pain and feelings of uncertainty, stress, and hopelessness. However, unlike our study, Ngo et al.^31^ reported some positive effects of chronic pain on family life, such as stronger relationships, personal growth, and a reformed view on life. It is also interesting to compare the results of our study with those of quantitative studies examining the impact of chronic pain on adolescents and their families. Past quantitative research shows that mothers of adolescents with chronic pain report restrictions in social life and difficulties coping with their child’s pain.^29^ Logan et al.^30^ found that parental pain catastrophizing and protective responses to child pain influence overall school functioning and absenteeism, highlighting how parental responses to a child’s chronic pain impact the child with pain.

Our study responds to several key gaps identified in the literature. Unlike many studies that focus on a single condition, ours integrates data from parents of adolescents with a broad range of chronic pain types, including headaches (67% of the sample), musculoskeletal pain, and abdominal pain, contributing to the scarcity of conceptually rich family-focused studies on these common conditions.^45^ By focusing on parents whose children are enrolled in a pain management program, we offer detailed insights into how families navigate treatment and services, such as pain management plans and social care interactions, thereby addressing the limited qualitative work on these processes.^45^ Our findings span both the day-to-day experience of living with pain and families’ interactions with health care systems, schools, and insurers, providing rare qualitative detail on service experiences,^45^ and the emphasis on siblings’ experiences, marital strain, and financial and logistical burdens underscores the need for family-centered interventions and outcomes. The inclusion of fathers and discussions of sibling dynamics help remedy the underrepresentation of these family members.^45^ Using open-ended questions ensured that parents’ responses emerged organically, capturing the depth and complexity of the physical, psychological, social, and school-related effects on adolescents alongside the emotional, practical, and relational impacts on families. Collectively, these insights update and extend the existing body of work and offer critical evidence to inform the design and delivery of health and social care services, interventions, and future research in pediatric and adolescent chronic pain.

### Clinical implications

Our findings reflect the biopsychosocial conceptualizations of pain as parents highlighted the psychological, physical, social, and school impacts on their child and family life. The importance of multidisciplinary approaches to adolescent chronic pain management is evident to address these areas of impact. A multidisciplinary program that provides comprehensive, coordinated care for adolescents with chronic pain under one roof would be optimal to ensure access to all therapeutic approaches. Person-centered physical activity and exercise interventions that are tailored to the adolescent’s needs, expectations, and abilities are an essential treatment component of any comprehensive pain management program. Pain psychology interventions aim to help a person cope with and build skills to personally manage the thoughts, feelings, and behaviors that accompany chronic pain. Importantly, multidisciplinary treatment programs need to consider barriers to access to care, such as financial, structural, and geographical. Integrating social work services into these programs can assist patients and families navigate school and work stressors as well as potential barriers to care.

The significant negative impact of chronic pain on family life reinforces the need for systematically integrating family-based interventions into adolescent chronic pain management. Family-based interventions should address the unique challenges and needs of families managing adolescent chronic pain and include psychosocial support and resource provision to empower families to support adolescents with chronic pain. Such interventions may also help foster an environment that supports adolescents to generalize skills outside of the treatment program to use in their daily lives. Parents of adolescents with chronic pain may also benefit from counseling to explore the difficult feelings, such as helplessness, guilt, and stress, brought about by their child’s chronic pain. Counseling for parents may be a powerful way to model positive behaviors for their children; for example, how to deal with emotions in a healthy way or adaptively cope with stressors or adversity. Moreover, programs that address the needs and feelings of siblings and other adult caregivers of adolescents with chronic pain may help create a better experience for all involved throughout the adolescent’s chronic pain journey. Pain clinics or programs should also consider adopting strategies to alleviate the potential burden of frequent appointments for chronic pain care. Pain clinics or programs could consider offering virtual appointments to adolescents with chronic pain or leveraging digital health technologies for at-home pain management to limit travel frequency.

Our findings highlight the potential utility of systematic psychosocial well-being screening for both adolescents with chronic pain and their families. Assessing psychosocial risk early in treatment will help identify those adolescents in need of additional resources and ongoing support to optimize psychosocial outcomes. Continued research in this area is essential to fully understand the impact of chronic pain on adolescents and their families and examine how these impacts differ according to individual factors, such as stage of development (age at diagnosis), medical factors (e.g., course of treatments), and environmental factors (e.g., social determinants of health). Understanding psychosocial issues experienced by adolescents and their families can inform the development of family-based interventions that promote adaptive coping, open and honest communication, resilience, and well-being. Web-based social support interventions may also be a promising strategy to improve emotional and social well-being in both adolescents with chronic pain and their families by enabling them to connect with others with similar experiences. Future research in this area is warranted. It is also important to acknowledge that adolescents with chronic pain and their parents often feel dismissed by health care professionals. Thus, it is imperative that health care professionals take time to build strong, trusting relationships with adolescents and their families. Health care professionals should show a genuine understanding of the adolescent’s unique experience and strive to foster a relationship in which the adolescent and their family feel heard, understood, and respected.

### Limitations and implications for future research

It is important to note the limitations of this study. First, our sample comprised mostly of mothers of daughters with chronic pain. Mothers are, however, more commonly the primary caregivers of adolescents with chronic pain.^37^ Chronic pain is also more common in teenage girls than boys.^88,89^ Second, we did not collect data on parents’ gender identity, religious, ethnic, cultural, or political backgrounds. Future research should collect this type of demographic information and explore whether, and how, these demographic characteristics influence the lived experiences of being a parent of an adolescent with chronic pain. Third, the participants were recruited through comprehensive pain services—programs offering a variety of services to meet the individualized needs of adolescents with chronic pain. As such, it is likely that the children of our participants experienced high-impact chronic pain that significantly affected their daily life and functioning. It is possible that parents’ accounts may have differed if parents of adolescents with lower-impact chronic pain were interviewed. Future qualitative studies garnering the perspectives of parents of adolescents with chronic pain treated in other types of settings would provide greater insight into the experiences of adolescents with chronic pain and their families.

Fourth, these interviews offer only a one-time retrospective account of these parents’ experiences and their perceptions of their child’s experiences. Longitudinal qualitative studies that explore how experiences evolve over time should be considered to better our understanding of chronic pain in adolescents, including their experiences transitioning from pediatric to adult chronic pain care. We interviewed parents of adolescents with chronic pain as opposed to adolescents with chronic pain themselves and thus it is possible that our findings do not accurately reflect the actual experiences of adolescents with chronic pain. We recommend future mixed methods studies that combine surveys and a dyad approach, wherein parent and child are interviewed together, to gain greater insight into the impact of chronic pain on adolescents and their families. Despite these limitations, our findings provide guidance for strategies to help adolescents and their families as they navigate the challenges of living with chronic pain.

## Conclusion

This study makes clear the profound impact of chronic pain on adolescents and their families. Importantly, these narratives support the need for strategies and interventions to mitigate the physical, psychological, and social impact of chronic pain on adolescents. Our findings also call for family level interventions and resources, such as resilience enhancement or coping interventions, to support families of adolescents with chronic pain. Interviewing adolescents with chronic pain would shed additional light on their needs and experiences and inform the structure of pain programs and development of interventions to reduce emotional distress, enhance coping skills, and improve quality of life in adolescents with chronic pain.

## Supplementary Material

Interview guide.docx
