# Supplementary material for: The impact of chronic pain on adolescents and their families: A qualitative investigation of parental perspectives
Source: Can J Pain. 2025 Dec 8;9(1):2562440. doi: 10.1080/24740527.2025.2562440 (PMC12688254; doi:10.1080/24740527.2025.2562440)
Supplement: Interview guide.docx [file UCJP_A_2562440_SM3297.docx]

# **A qualitative exploration of the views and experiences of children and adolescents with chronic pain**

*“Hello, my name is ___________________________ and I represent the research team at The Ohio State University and Nationwide Children’s Hospital. Thank you so much for your time. The purpose of this interview is to explore the views and experiences of children and adolescents with chronic pain from the perspective of their parents. Chronic pain is any pain that lasts for three months or more. You are selected to participate in this study because you have a child with chronic pain.*

*The interview should take approximately 30 minutes to complete. You will be compensated $25 for your participation in the interview. There are no right or wrong answers. You may skip any of the questions you do not want to answer. Your responses will be audio recorded so there is a complete record of what was said during the interview, and so I do not have to write down all your responses as we are talking. You may disagree to the audio recording. Your responses will remain strictly confidential, and you will not be identified by name in any report or publication. Do you agree to the audio recording? Do you have any questions before we begin?* [Answer questions]. *Great! Let’s begin the interview.*

**section 1: HISTORY OF CHRONIC PAIN**

**Tell me a little bit about when your child first started having chronic pain.**

- *Prompts:* How did you find out your child had chronic pain? What symptoms did your child have? What thoughts/feelings did you have at the time of your child’s diagnosis? Before your child was diagnosed with chronic pain, had you heard of the condition? How long did it take for your child to get a diagnosis?

**What were you told about chronic pain when your child first sought care for their pain?**

- *Prompts:* Can you explain how chronic pain was explained to you? What were you told about the treatment of your child’s chronic pain? What do you remember as being helpful when you were getting all this information? What do you remember as being unhelpful when you were getting this information?

**SECTION2: EXPERIENCES WITH CHRONIC PAIN**

**Tell me a little about your child’s experiences since they first started having chronic pain.**

- *Prompts:* How do you feel chronic pain has affected, if at all, aspects of your child’s life? Are there things that are different for your child now (e.g., school, activities)? How has your child coped with their chronic pain? What has your child found to be most helpful since the time of their diagnosis, if anything?

**Can you tell me a little bit about your experiences since your child first started having chronic pain? What about the rest of your family?**

- *Prompts:* Think about the last month or so. How do you juggle the demands of caring for your child, being a parent, and living your life? What areas of your family life have been affected by your child’s chronic pain, if any? What, if any, have been the difficulties you and your family have had to deal with since the time of your child’s diagnosis? What have you found to be most helpful to you or your family since the time of your child’s diagnosis, if anything?

**SECTION 3: MANAGEMENT OF CHRONIC PAIN**

**How is your child’s chronic pain treated?**

- *Prompts:* What type of services has your child received? For example, have they received medication management, physical and/or occupational therapy, chiropractic, or counselling services? Can you tell me a little bit about the services they received? Were these services specific to pain management? What types of treatments have been most successful? What types of treatments have been least successful? Has your child participated in any special pain programs? If so, can you tell me about these programs?

**What strategies or techniques does your child use to manage their pain?**

- *Prompts:* How is your child’s pain managed? When your child has pain, what do they do? Who do they tell? Generally, how does your child react when they are in pain?

**SECTION 4: VR interventions for chronic pain management**

*We are interested in hearing your thoughts on a Virtual Reality (VR) program to help children manage their chronic pain on a daily basis. We’d like to understand what parents like you think about this possibility.*

**Do you think a VR program that helps your child manage their chronic pain at home would be useful? Why or why not?**

**Has your child ever used a VR program or application such as a mobile app or web-based platform to help them manage their pain?**

____Yes_____No.

- *Prompts:* If yes, which ones? Can you please tell me a little bit about the VR program or application your child has used? How often did they use these program(s)? What did your child like about the program(s) they used? What didn’t they like about the programs they used? How can these programs be improved?

**If you were to create/design a VR program for children with chronic pain, what would it look like?**

- *Prompts:* What would you expect from the program? How would it be used by children? What kind of information do you think children need in a VR program to help manage their chronic pain at home? How should this information be presented? What tools would be useful for at home management of their chronic pain? What types of features would the program include?

**What are potential barriers, if any, to VR program use among children with chronic pain? Would anything promote the use of a VR program among children with chronic pain?**

- *Prompts:* What challenges and opportunities exist for VR programs for the management of chronic pain in children? How can we overcome these barriers/challenges? How could we encourage children with chronic pain to use a VR program to manage their pain?

**SECTION 5. Concluding questions**

**Suppose a group of health-care staff were trying to decide the best way to support children with chronic pain, what would you recommend?**

**Is there anything else that you’ve learned through your experiences that you think would help children with chronic pain and their families?**

**Is there anything else that you want to add?**

# This is the end of the interview. Thank you so much for your time and participation. Your comments were extremely helpful.
